# Chronic Administration of Pimozide Fails to Attenuate Motor and Pathological Deficits in Two Mouse Models of Amyotrophic Lateral Sclerosis

**DOI:** 10.1007/s13311-018-0634-3

**Published:** 2018-05-22

**Authors:** Silvia Pozzi, Sai Sampath Thammisetty, Jean-Pierre Julien

**Affiliations:** 1 CERVO Brain Research Center, 2601 Chemin de la Canardière, Québec, Québec G1J 2G3 Canada; 2 Department of Psychiatry and Neuroscience, Faculty of Medicine, Université Laval, Québec City, G1V 0A6 Canada

**Keywords:** Pimozide, ALS, SOD1, TDP-43

## Abstract

Amyotrophic lateral sclerosis (ALS) is a fatal neurodegenerative disease which presently does not have any efficient therapeutic approach. Pimozide, a Food and Drug Administration (FDA)-approved neuroepileptic drug, has been recently proposed as a promising treatment for ALS patients based on apparent stabilization of right hand muscles after a short-time administration. A new clinical trial started at the end of 2017 to recruit patients with a prolonged drug delivery schedule. Here, our aim was to investigate the effects of chronic administration of pimozide on disease progression and pathological events in two mouse models of ALS. Pimozide was administered every 2 days to transgenic mice bearing the ALS-linked A315T mutation on the human TAR DNA-binding protein 43 (TDP-43) gene and to mice carrying the human superoxide dismutase 1 (SOD1) gene with the ALS-linked G93A mutation. Chronic administration of pimozide exacerbated motor performances in both animal models and reduced survival in SOD1^G93A^ mice. In TDP-43^A315^T, it decreased the percentage of innervated neuromuscular junctions (NMJs) and increased the accumulation of insoluble TDP-43. In SOD1^G93A^ mice, pimozide had no effects on NMJ innervation or motoneuron loss, but it increased the levels of misfolded SOD1. We conclude that a chronic administration of pimozide did not confer beneficial effects on disease progression in two mouse models of ALS. In light of a new clinical trial on ALS patients with a chronic regime of pimozide, these results with mouse models suggest prudence and careful monitoring of ALS patients subjected to pimozide treatment.

**Electronic supplementary material:**

The online version of this article (10.1007/s13311-018-0634-3) contains supplementary material, which is available to authorized users.

## Introduction

Amyotrophic lateral sclerosis (ALS) is a fatal neurodegenerative disease which affects motor neuron health leading to muscle paralysis and ultimately death after 3–5 years from symptoms onset. Ninety percent of cases have a sporadic etiology, whereas 10% show a genetic inheritance but no difference can be observed in symptoms onset and progression between the two disease types [1]. The discovery of different genes involved in the pathogenesis of familial cases allowed the generation of animal models for the study of disease mechanisms and the test of therapeutic strategies [2]. Although treatments for ALS have been approved, their efficiency remains mild; therefore, new therapeutic strategies have to be still discovered and proposed to cure this lethal disease [1]. Pimozide has been recently suggested as a new potential treatment for ALS [3].

Pimozide is a Food and Drug Administration (FDA)-approved neuroepileptic compound mainly used for schizophrenia [4], delusional disorder syndrome [5], and tics [6, 7]. At the molecular level, it has a specific dopamine blocking activity (D2 receptor antagonist with high affinity for 5-HT7 receptor) and a T-type Ca^2+^ channel inhibitory effect [8]. Pimozide was first tested on ALS patients in 1998 [9] and recently emerged as potential treatment for this disease with beneficial effects at the level of neuromuscular junctions (NMJs) [3]. Indeed, after a large screening of molecules in *Caenorhabditis* *elegans*, pimozide appeared as the most effective compound in stabilizing motor functions and in maintaining NMJ structure and synaptic transmission in mutant TDP-43 zebrafish models. This study showed that pimozide was able to enhance the synaptic transmission of NMJs also in SOD1^G37R^ mice. A phase II randomized controlled (RCT) trial was then carried out for 6 weeks in ALS patients, and it yielded preliminary evidence of beneficial effect of pimozide on NMJs function and preservation of the decremental muscles in right hand [3].

Although promising, the studies carried out by Patten et al. [3] were all performed as acute treatments. *C. elegans* and zebrafish models underwent a 12–16-h treatment, ex vivo NMJs models from SOD1^G37R^ mice were continuously perfused for a period of 20–45 min, and patients received a 6-week treatment. Here, we investigated the effects of chronic administration of pimozide in two mouse models of ALS, TDP-43^A315T^ mice [10], and SOD1^G93A^ mice [11]. We administered 1 mg/kg of pimozide intraperitoneally every 2 days and analyzed the motor performance and survival of mice. According to the guide for dose conversion from human to mouse [12], we chose a dosage for mice treatment meant to be intermediate between the two doses used for patient trial [3]. Microscopy and biochemical analyses were carried out to assess the effects of pimozide on NMJ denervation and to pathological features that characterize ALS, including TDP-43 cytoplasmic mislocalization/accumulation and SOD1 misfolding. Our results revealed that long-term treatment exacerbated motor phenotypes and proteinopathy in the two mouse models of ALS. We conclude that pimozide should be administered cautiously in ALS patients especially in context of prolonged treatment.

## Methods

### Animals

Transgenic mice expressing human TDP-43^A315T^ were generated and characterized previously [10]. Mice overexpressing human SOD1^G93A^ (B6SJL-TgN_[SOD1-G93A]_1 Gur) were purchased from the Jackson Laboratory. Lines were maintained in heterozygosity in the C57BL/6 background. All experimental procedures were approved by the Laval University Animal Care Ethics Committee and are in accordance with the Guide to the Care and Use of Experimental Animals of the Canadian Council on Animal Care. Brains, spinal cords, and tibialis muscles of all animals were collected for biochemical or histopathological analyses after anesthesia. SOD1^G93A^ mice were considered end-stage with extension reflex scoring 0 and inability to turn themselves in 30 s when lying on one side.

### Pimozide Treatment

Pimozide (Sigma) was prepared for injection with 5% DMSO (dimethyl sulfoxide, Sigma), 2% Tween-20 (Sigma) in saline at the concentration of 0.18 mg/ml. Pimozide and vehicle solution were administered every 2 days by intraperitoneal (i.p.) injection at the dose of 1 mg/kg. TDP-43^A315T^ mice were injected i.p. at 9 months of age with pimozide (*n* = 12, females *n* = 5, males *n* = 7) or vehicle (*n* = 8, females *n* = 6, males *n* = 2) and followed for 4 months. SOD1^G93A^ female mice were injected starting at 50 days of age with pimozide (*n* = 16) or vehicle (*n* = 15) or at 90 days of age with pimozide (*n* = 16) or vehicle (*n* = 15) and followed until end-stage. Three to four SOD1^G93A^ mice per group were euthanized for histopathological studies at symptomatic stage (120 days of age).

### Behavioral Tests

Once per week, animals underwent rotarod test, grip strength test, and weight measure. Mice were trained 1 week before treatment, and data were collected from the beginning of the treatment for TDP-43^A315T^ mice and SOD1^G93A^ treated from 90 days of age and starting from 70 days of age for SOD1^G93A^ mice treated from 50 days of age. Rotarod (Ugo Basile) was performed at 4-rpm/s speed and 0.25-rpm/s or 0.1-rpm/s acceleration for TDP-43^A315T^ and SOD1^G93A^ mice, respectively. Time (s) before falling from the bar was noted, and best performance out of three tests was considered for analyses. Grip strength was evaluated by grid test. Briefly, animals were positioned on a grid which was turned up-side-down. Time (s) taken before falling from the grid was considered for analyses with a maximum time of 90 s. Extension reflex was evaluated in SOD1^G93A^ mice using a 3-point score system from 3 (hind limbs extending to form an angle of 120°) to 0 (loss of reflex with hind limbs paws held close to the body and inability to walk).

Tremors were evaluated on a 4-point score system where 0 was identified as no tremors and 4 as strong tremors in hind limbs or mild tremors in fore and hind limbs.

### Immunofluorescence

After anesthesia, mice were intracardially perfused with phosphate-buffered saline followed by 4% PFA (Paraformaldehyde, Sigma). Tissue samples were then post-fixed overnight in 4% PFA and subsequently equilibrated in 30% sucrose. Tissues were cut in 25-μm transverse sections on a Leica frozen microtome and kept in a cryoprotective solution at − 20 °C. Sections were washed in TBS and permeabilized in TBS-T (Triton 0.2%, Sigma) before blocking in 10% goat serum in TBS-T for 1 h at room temperature. Primary antibodies, mouse monoclonal anti-hTDP-43 (1:500, ABNOVA), rabbit polyclonal anti-neuronal nuclear marker (NeuN, 1:500, Cell Signaling), rabbit polyclonal anti-Iba1 (1:500, ionized calcium-binding adapter molecule 1, WAKO), and mouse monoclonal anti-misfolded SOD1 B8H10 (1:50, in house made [13]), were incubated overnight at room temperature. After washes, signal was revealed by incubation with Alexa Fluor (Thermo Fisher Scientific) secondary antibodies 1:500 in blocking buffer, 2 h at room temperature, and nuclei were counterstained with Dapi 1:1000 (Invitrogen) for 1 min followed by washes. Sections mounted on slides were closed with Fluoromount-G Buffer (Southern Biotech). Images were acquired using confocal microscope (Olympus), and signal quantification was performed by ImageJ software (NIH, Bethesda, MD) [10, 13, 14]. Nuclear and cytoplasmic TDP-43 was analyzed as previously described [14]. Briefly, the integrated density of nuclear TDP-43 (identified by Dapi staining) and of total TDP-43 (identified by NeuN) was obtained with ImageJ per each cell observed in the picture. Cytoplasmic TDP-43 was obtained by subtraction and nuclear/cytoplasmic ratio was subsequently considered. Nissl staining was performed on slide-mounted sections. Slides were dehydrated by 5 min incubations in water, 70% ethanol, 90% ethanol, 95% ethanol, and 100% ethanol and rehydrated in same but descending series of alcohol till water. One percent cresyl violet staining was performed for 3 min followed dehydration steps as previously performed. Slides were closed using DPX mounting medium (Sigma). Images were acquired at ×10 using Apotome microscope (Carl Zeiss) and analyzed for motoneurons larger than 200, 250, or 300 μm^2^ using ImageJ software in a selected area including only the gray matter of the ventral horns. Misfolded SOD1 was quantified as integrated density of B8H10 antibody signal by ImageJ software in areas covering the gray matter of the ventral horns of lumbar spinal cords. NMJs were counted on tibialis muscles. One muscle per mouse was serial sectioned at 25 μm on slides. One slide, representing the entire muscle, was washed three times in PBS and blocked 30 min in PBS, 10% normal goat serum, and triton 0.25%. Nerve fibers were stained with rabbit anti-PGP9.5 antibody (Protein Gene Product 9.5, Serotech) 1:500 overnight at room temperature in PBS. After washes, anti-rabbit Alexa Fluor-488 (Thermo Fisher Scientific) secondary antibody 1:500 was incubated 2 h at room temperature in PBS together with anti-Bungarotoxin (Btx)-Rodamine conjugated 1:1000 (Sigma). Slides were closed after washes with Fluoromunt-G buffer (Southern Biotech). Images were acquired using confocal microscope (Olympus). All post-synaptic NMJs Btx-positive were counted in each slide containing 8–10 serial muscle sections, and complete, partial, or totally absent overlapping with PGP9.5 staining was considered for discriminating innervation.

### Biochemical Analyses

Brain cortices or lumbar spinal cords were processed to obtain soluble and insoluble fractions as follows. Tissues were homogenized in 50-mM Tris-HCl pH 7.4, 100-mM NaCl, 10% glycerol, 1% Triton X, and protease inhibitors cocktail. Lysates were rotated for 30 min at 4 °C and then centrifuged for 20 min at 9000 ×*g*. Supernatants were used as soluble fraction; pellet was sonicated in 6-M urea and 3% SDS (sodium dodecyl sulfate) buffer. Urea lysates were centrifuged at 15,000 ×*g* for 20 min at 4 °C and the supernatants collected as insoluble fraction. Fifteen micrograms of proteins was loaded and resolved in 12% polyacrylamide SDS-PAGE gel and transferred on PVDF membranes (Immobilon-P, Millipore). After transfer, membranes were stained by Ponceau (Sigma) for total transferred protein evaluation. Western blot was performed by blocking in 3% BSA (bovine serum albumin, Biobasic Canada) prepared in TBS-tween 1% (VWR Lifescience). Proteins of interest were detected by incubating the membrane overnight in blocking buffer with primary antibodies: mouse monoclonal anti-hTDP-43 (1:1000, Abnova) and rabbit polyclonal anti-GFAP (1:5000, glial fibrillary acidic protein, DAKO). Finally, membranes were incubated with respective horseradish peroxidase secondary antibodies 1:5000 in blocking buffer, and chemiluminescence was revealed by ECL reagent (electrogenerated chemiluminescence, Thermo Fisher Scientific) on light films (Kodak). Immunoreactivity was quantified by Image Lab software (Biorad) and normalized on Ponceau signals.

### Statistical Analyses

Statistical analyses were performed using Prism 5.0 software (GraphPad Software, LaJolla, CA, USA). Statistical test used for each analysis is mentioned in the text; *p* < 0.05 was considered statistically significant.

## Results

### Pimozide Treatment Reduced Motor Performances of TDP-43^A315T^ and SOD1^G93A^ Mice

**Fig. 1:**
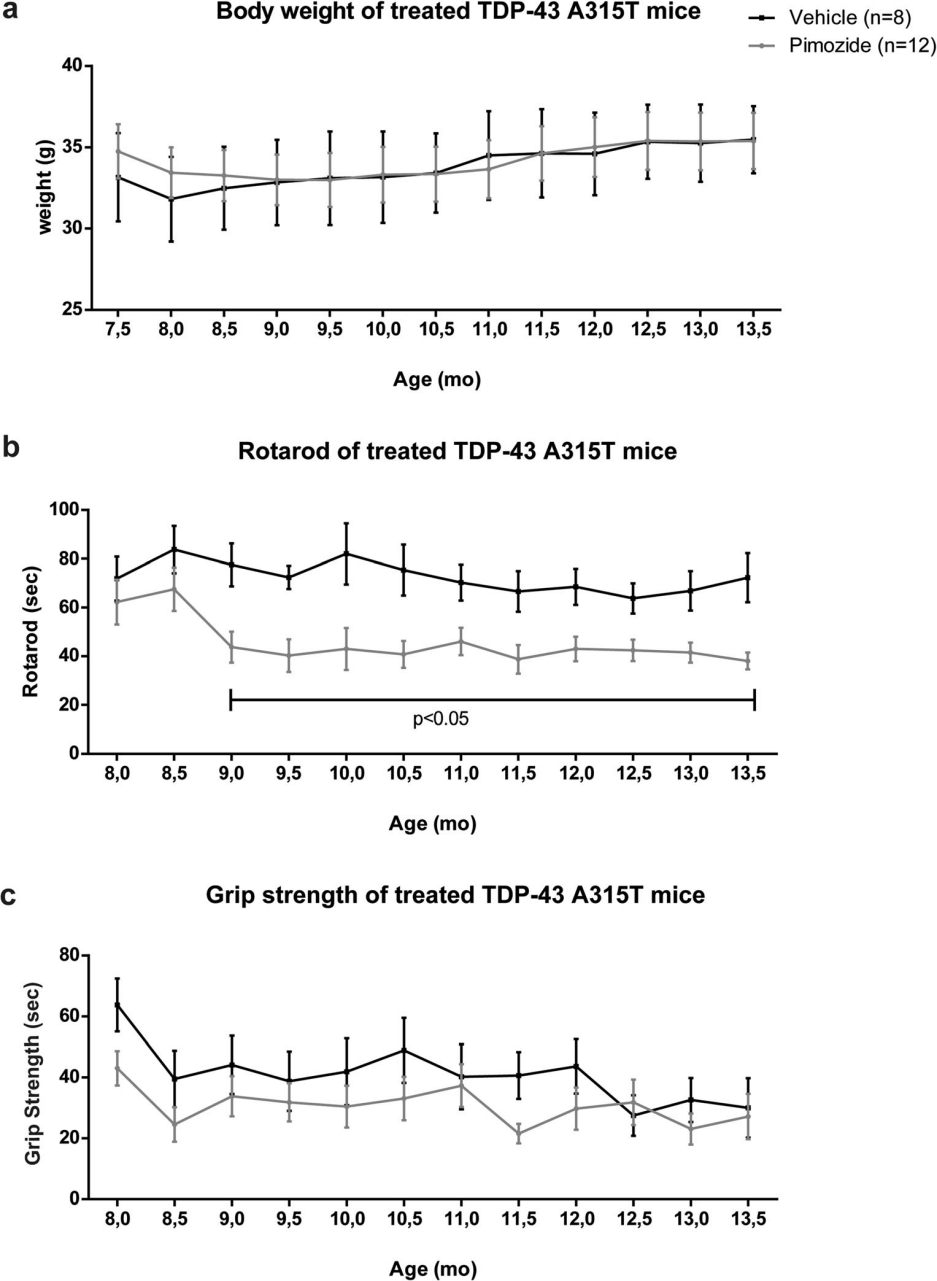
Pimozide treatment exacerbated motor performance in TDP-43^A315T^ mice. TDP-43^A315T^ mice were treated at 9 months of age with intraperitoneal injection of 1-mg/kg pimozide (*n* = 12) or vehicle (*n* = 8) every 2 days and followed for 4 months. Data were analyzed by two-way ANOVA followed by uncorrected Fisher’s LSD post hoc test. (**a**) Body weight analysis of animals showed no differences between treatments (two-way ANOVA analysis: interaction *p* > 0.05, time *p* < 0.0001, treatment *p* > 0.05). (**b**) Rotarod test revealed significant differences between pimozide- and vehicle-injected mice starting from the beginning of the treatment (two-way ANOVA analysis: interaction *p* > 0.05, time *p* > 0.05, treatment *p* < 0.0001). (**c**) No significant differences were observed for grip strength test (two-way ANOVA analysis: interaction *p* > 0.05, time *p* < 0.0001, treatment *p* > 0.05). Data are expressed as mean ± sem of percentage of maximum performance for each single mouse

The TDP-43^A315T^ and SOD1^G93A^ mice were chronically injected at a dose of 1-mg/kg pimozide or vehicle every 2 days. Figure 1 shows changes in body weight and motor performances of TDP-43^A315T^ mice subjected to pimozide treatment starting at 9 months of age for a 4-month-period (*n* = 12 for pimozide and *n* = 8 for vehicle). The drug treatment did not affect the body weight (Fig. 1a). Rotarod test (Fig. 1b) provided evidence that the ability of walking on a rotating bar of TDP-43^A315T^mice treated with pimozide was significantly impaired when compared to vehicle-treated mice. The grip strength of pimozide-treated mice was also slightly reduced when compared to vehicle-treated mice, and this change was significant according to two-way ANOVA (Fig. 1c).

**Fig. 2:**
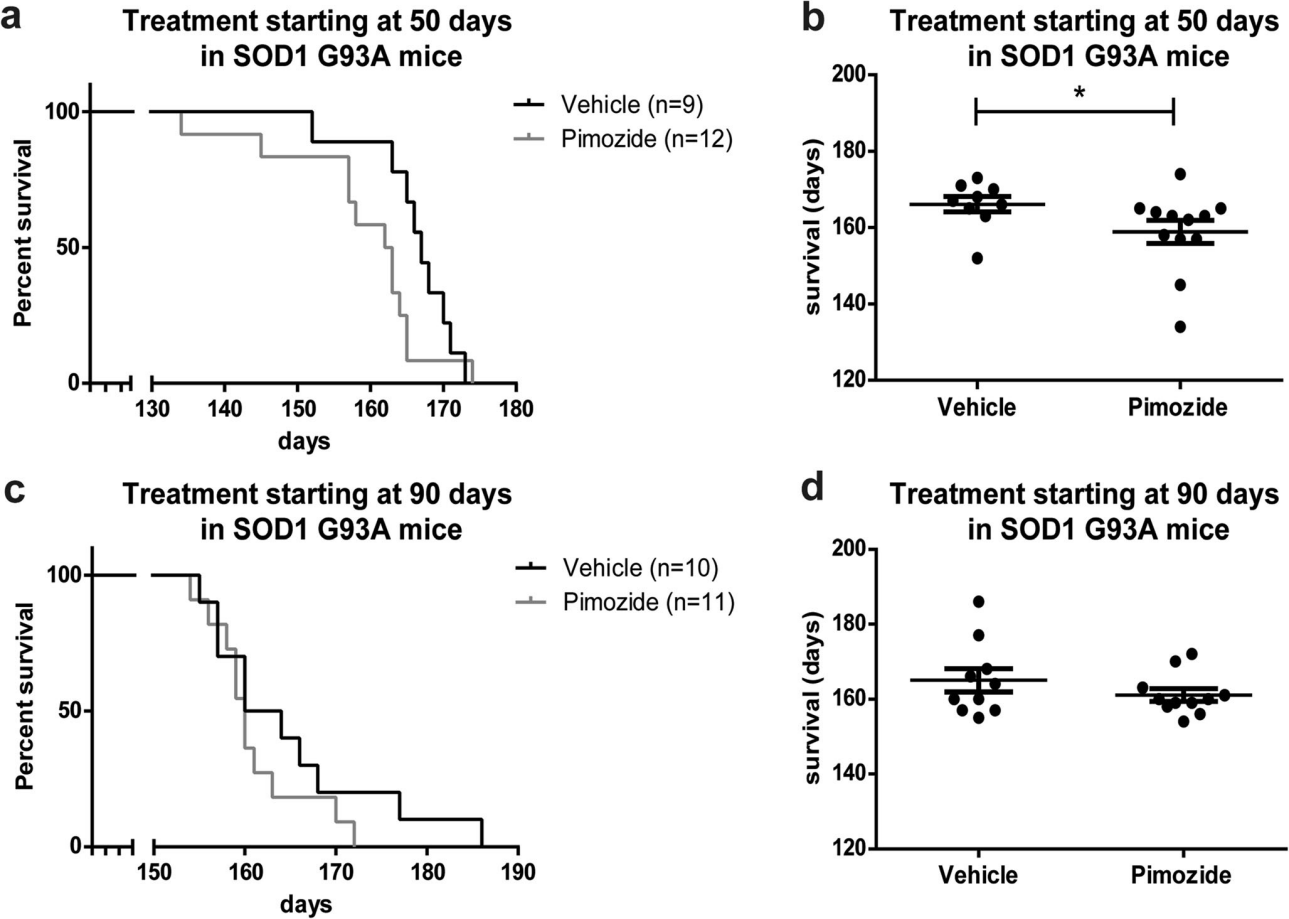
Pimozide treatment reduced lifespan of SOD1^G93A^ mice. (**a**, **b**) SOD1^G93A^ mice were treated from 50 days of age (*n* = 12 for pimozide, *n* = 9 for vehicle) or (**c**, **d**) from 90 days of age (*n* = 11 for pimozide, *n* = 10 for vehicle) with intraperitoneal injection of 1-mg/kg pimozide or vehicle every 2 days and followed till end-stage Survival curves (**a**, **c**) and dot graphs (**b**, **d**) of average survival of SOD1^G93A^ mice show a significant detrimental effect of pimozide treatment when administered from 50 days of age (mean 158.9 ± 3.0 days for pimozide and 166.1 ± 2.0 days for vehicle, *p* = 0.027 by Wilcoxon test and *p* = 0.026 by Mann-Whitney test), whereas when administered from 90 days of age the average survival is not affected (161.1 ± 1.6 days for pimozide and 165 ± 3.1 days for vehicle, *p* > 0.05 by Wilcoxon test and Mann-Whitney test); data are mean ± sem in days.

**Fig. 3:**
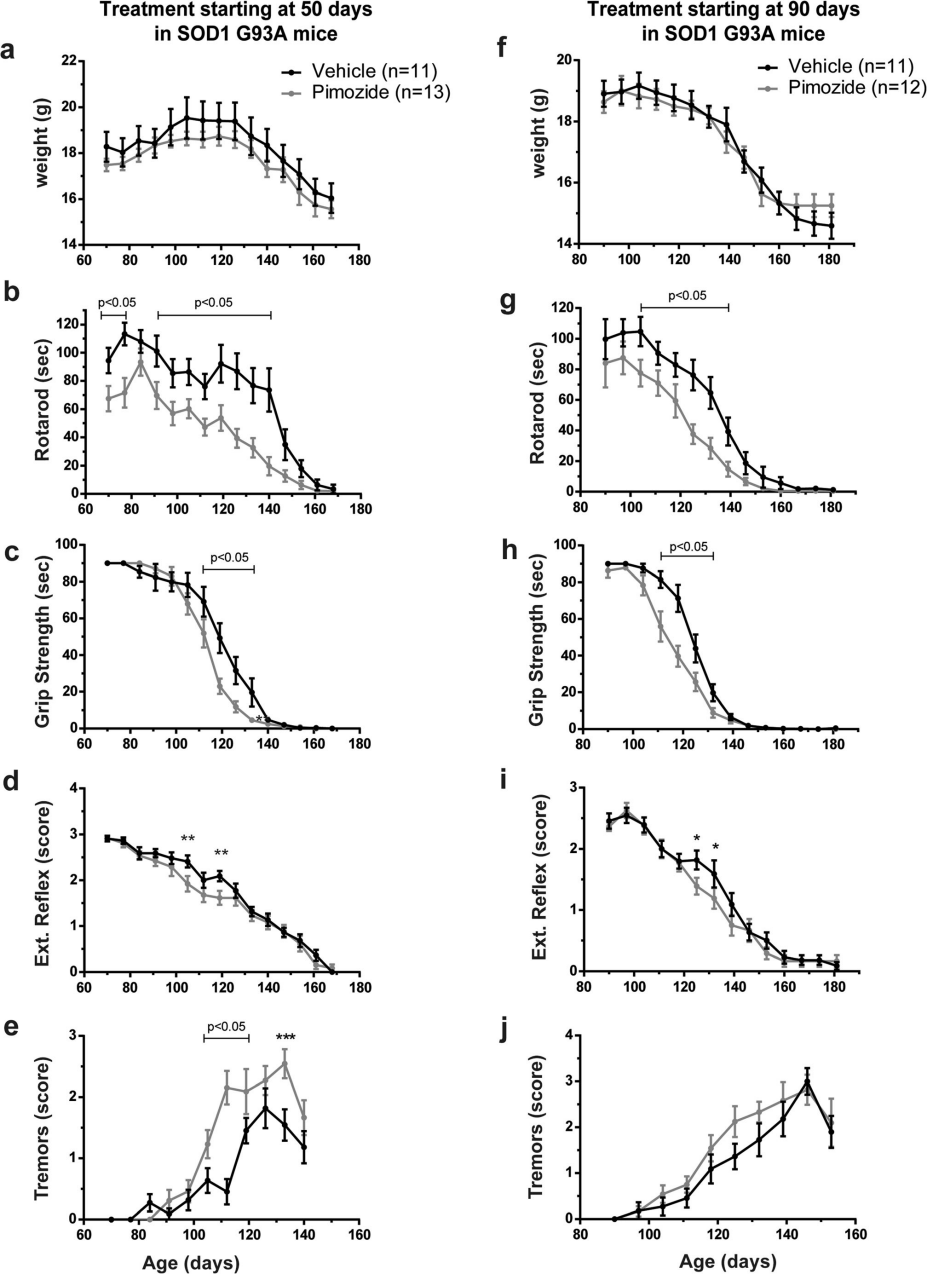
Deleterious effects of pimozide on motor performance of SOD1^G93A^ mice. (**a**–**e**) SOD1^G93A^ mice were treated from 50 days of age (*n* = 13 for pimozide, *n* = 11 for vehicle) or (**f**, **j**) from 90 days of age (*n* = 12 for pimozide, *n* = 11 for vehicle) with intraperitoneal injection of 1-mg/kg pimozide or vehicle every 2 days and followed till end-stage. Data were analyzed by two-way ANOVA followed by uncorrected Fisher’s LSD post hoc test. (**a**, **f**) No significant differences were observed for body weight (two-way ANOVA analysis: interaction *p* > 0.05, time *p* < 0.0001, treatment *p* = 0.0018 for treatment starting at 50 days and interaction *p* > 0.05, time *p* < 0.0001, treatment *p* > 0.05 for treatment starting at 90 days of age). (**b**, **g**) Rotarod test results are significantly different from 70 to 140 days of age in animals treated from 50 days of age (two-way ANOVA analysis: interaction *p* > 0.05, time *p* < 0.0001, treatment *p* < 0.0001) and from 104 to 139 days of age in animals treated at 90 days of age (two-way ANOVA analysis: interaction *p* > 0.05, time *p* < 0.0001, treatment *p* < 0.0001). (**c**, **h**) Grip strength was significantly different with marked differences from 112 to 133 days for both treatments (two-way ANOVA analysis: interaction *p* = 0.0005, time *p* < 0.0001, treatment *p* = 0.0006 for the treatment starting at 50 days and interaction *p* < 0.0001, time *p* < 0.0001, treatment *p* < 0.0001 for the treatment starting at 90 days). (**d**, **i**) Extension reflex was different between treatments with significant values from 105 to 120 days of age for animals treated at 50 days of age and from 125 to 132 days of age for animals treated at 90 days of age (two-way ANOVA analysis: interaction *p* > 0.05, time *p* < 0.0001, treatment *p* = 0.0019 for treatment starting at 50 days of age and interaction *p* > 0.05, time *p* < 0.0001, treatment *p* = 0.0452 for treatment starting at 90 days of age). (**e**, **j**) We observed more tremors from 105 to 133 days when pimozide treatment started at 50 days of age, whereas no significant changes were observed when treatment started at 90 days of age (two-way ANOVA analysis: interaction *p* < 0.001, time *p* < 0.0001, treatment *p* < 0.001 for treatment starting at 50 days of age and interaction *p* > 0.05, time *p* < 0.0001, treatment *p* = 0.0283 for treatment starting at 90 days of age). Data are expressed as mean ± sem

Figure 2 shows that survival of SOD1^G93A^ mice receiving chronic pimozide treatment starting at 50 days of age (*n* = 12 for pimozide, *n* = 9 for vehicle) was shortened by 7 days when compared to vehicle-treated mice (Fig. 2a, b). No significant changes occurred when treatment started at 90 days of age (*n* = 11 for pimozide, *n* = 10 for vehicle) (Fig. 2c, d). The body weight of SOD1^G93A^ mice was not affected by pimozide treatment (Fig. 3a, f), whereas motor performances were exacerbated by pimozide. Mice receiving treatment at 50 days of age exhibited deficits in rotarod performance at the beginning of data collection, i.e., 70 days of age (Fig. 3b). The ability to walk on the bar was aggravated by pimozide treatment at symptomatic stages. Moreover, at symptomatic stage, there were significant differences in both grip strength (Fig. 3c) and extension reflex (Fig. 3d) between pimozide- and vehicle-treated mice. Finally, an increase of tremors in pimozide-treated mice (Fig. 3e) was also detected. In the same way, mice receiving treatment lately in the progression (at 90 days of age) exhibited a worse performance in rotarod test, grip strength, and extension reflex (Fig. 3g–i). In this group of mice, there was a slight increase of tremors in pimozide-treated SOD1^G93A^ mice compared to vehicle-treated mice (Fig. 3j).

### Loss of Neuromuscular Junctions in TDP-43^A315T^ and SOD1^G93A^ Mice Was Exacerbated by Chronic Pimozide Treatment

**Fig. 4:**
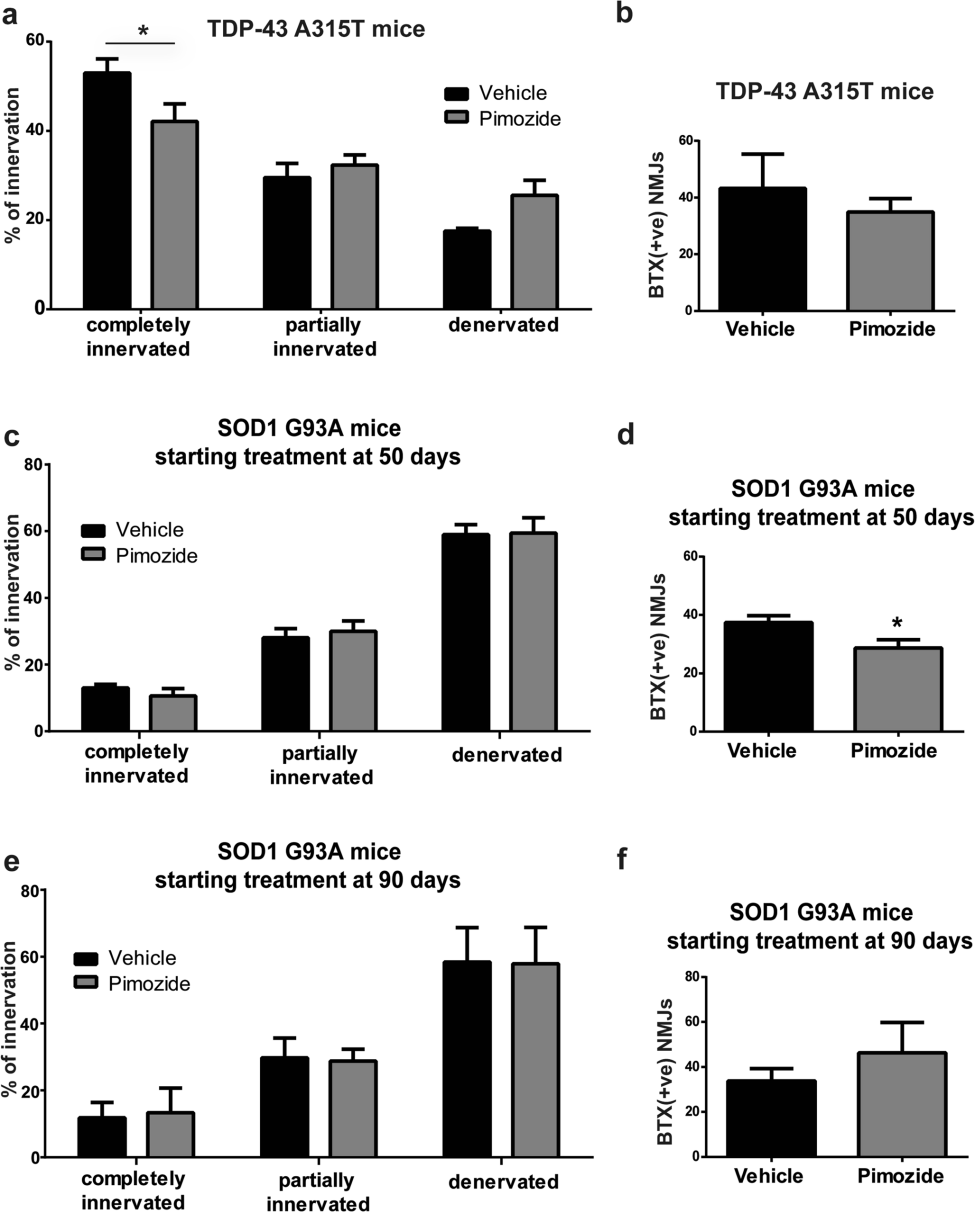
Pimozide treatment did not improve neuromuscular junction (NMJ) integrity in ALS mice. (**a**, **b**) Tibialis muscles of TDP-43^A315T^ (*n* = 4 per group, 2 females and 2 males) were analyzed for neuromuscular junction integrity. An average of eight sections per mouse was analyzed; data are expressed as mean ± sem of NMJ/section/mouse. Pimozide reduced the percentage of completely innervated NMJ compared to vehicle-treated mice (two-way ANOVA analysis: interaction *p* = 0.0146, innervations *p* < 0.0001, treatment *p* > 0.05 followed by uncorrected Fisher’s LSD post hoc test). No differences were observed in the total number of post-synaptic NMJ (data are mean ± sem of BTX + ve NMJ/section/mouse, *p* > 0.05 by Student *T* test). (**c**, **d**) Tibialis muscles of SOD1^G93A^ treated from 50 days of age (*n* = 3 per group) were analyzed at 120 days of age for neuromuscular junctions integrity. An average of nine sections per mouse was analyzed; data are expressed as mean ± sem of NMJ/section/mouse. No differences were observed in NMJ innervation/denervation (two-way ANOVA analysis: interaction *p* > 0.5, innervations *p* < 0.0001, treatment *p* > 0.05 followed by uncorrected Fisher’s LSD post hoc test). Total number of post-synaptic NMJ was instead reduced in pimozide-treated mice (data are mean ± sem of BTX + ve NMJ/section/mouse, *p* = 0.0387 by Student *T* Test). (**e**, **f**) Tibialis muscles of SOD1^G93A^ treated from 90 days of age (*n* = 3 per group) were analyzed at 120 days of age for neuromuscular junctions integrity. An average of nine sections per mouse was analyzed; data are expressed as mean ± sem of NMJ/section/mouse. No differences were observed in NMJ innervation/denervation (two-way ANOVA analysis: interaction *p* > 0.5, innervations *p* < 0.0001, treatment *p* > 0.05 followed by uncorrected Fisher’s LSD post hoc test) nor in total number of post-synaptic NMJ (data are mean ± sem of BTX + ve NMJ/section/mouse, *p* > 0.05 by Student *T* test)

NMJs were analyzed in the tibialis muscles of TDP-43^A315T^ mice at the end of the treatment (Fig. 4a, b) and in SOD1^G93A^ mice at 120 days of age (symptomatic stage) (Fig. 4c–f). Pimozide reduced the number of completely innervated NMJs in TDP-43^A315T^ mice (Fig. 4a) without altering the number of total post-synaptic junctions (Fig. 4b). At the symptomatic stage (120 days of age) in SOD1^G93A^ mice that received treatment from 50 days of age, pimozide did not affect the proportion of innervated and denervated NMJs (Fig. 4c); however, it reduced the number of post-synaptic NMJs (Fig. 4d). No changes occurred when mice were treated from 90 days of age (Fig. 4e, f).

### Pimozide Treatment Did Not Attenuate Pathology in TDP-43^A315T^ Mice

**Fig. 5:**
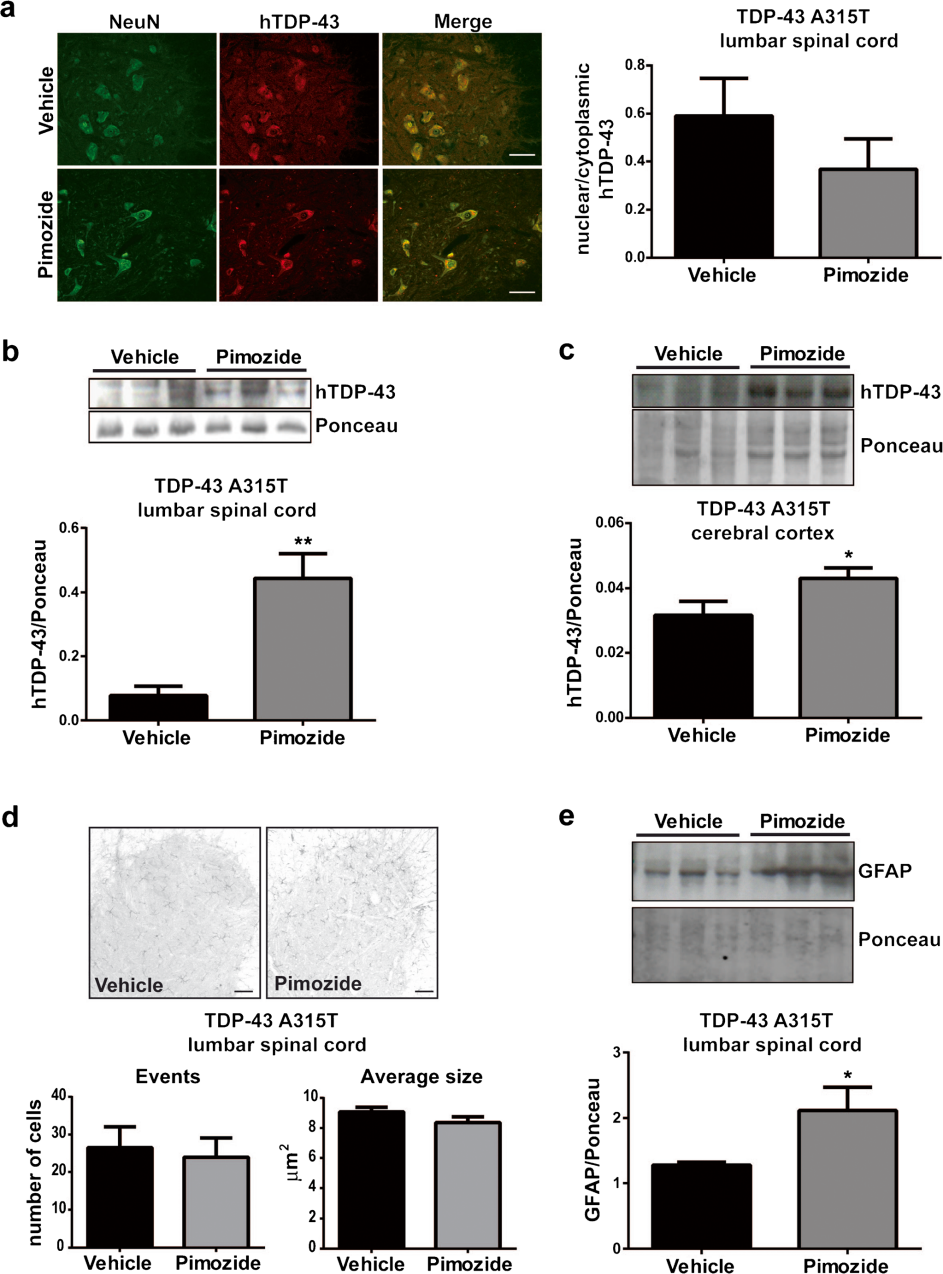
Pimozide treatment did not improve TDP-43 pathology of TDP-43^A315T^ mice. (**a**) Representative picture of human TDP-43 (red) and NeuN (green) in lumbar spinal cord sections. Nuclear/cytoplasmic localization of hTDP-43 was analyzed in 10 sections per mouse (*n* = 4 mice per group, two females and two males), and quantification is shown in the graph. No differences were observed in the nuclear localization of human TDP-43 after pimozide treatment (*p* = 0.312 by unpaired *T* test); data are mean ± sem, scale bar 50 μm. (**b**, **c**) Insoluble human TDP-43 was analyzed in lumbar spinal cord and cerebral cortex by western blot and normalized on Ponceau staining, i.e., total transferred proteins on the membrane, western blot, and quantification are shown. Pimozide treatment increased insoluble human TDP-43 in (**b**) lumbar spinal cord (*n* = 3 female mice per group, *p* = 0.010 by unpaired *T* test) and in (**c**) cerebral cortex (*n* = 3 female mice per group, *p* = 0.050 by Student *T* test); data are mean ± sem. (**d**) Representative picture of Iba1 staining in lumbar spinal cord. Number of events (cells/section) and body size of the cell were analyzed by ImageJ on 10 sections per mouse (*n* = 4 mice per group, two females and two males), and quantification is shown in the graph. No differences were observed in microglial activation after pimozide treatment (*p* = 0.749 for events and *p* = 0.196 for body size by unpaired *T* test), data are mean ± sem, scale bar_=_50 μm. (**e**) Astrocyte activation was analyzed by western blot in soluble fraction of lumbar spinal cord and normalized on Ponceau staining; western blot and quantification are shown. Pimozide treatment increased GFAP protein levels (*n* = 3 female mice per group, *p* = 0.038 by Student *T* test); data are mean ± sem

The TDP-43^A315T^ mice exhibit during aging TDP-43 mislocalization and aggregation in large motor neurons of the lumbar spinal cord and in cortical neurons [10]. As shown in Fig. 5a, pimozide treatment was unable to prevent cytoplasmic mislocalization of human TDP-43 in large NeuN positive cells of the lumbar spinal cord. We analyzed the levels of insoluble human TDP-43 in samples (*n* = 3) of lumbar spinal cord (Fig. 5b) and cerebral cortex (Fig. 5c). The results indicate that pimozide treatment led to an increase of human TDP-43 in the insoluble fractions of brain and spinal cord. We also investigated the levels of inflammation markers in TDP-43^A315T^ mice after 4 months of treatment. Pimozide treatment did not affect the number and size of Iba1-positive cells (Fig. 5d) suggesting no effects on microglial activation in spinal cord. However, pimozide enhanced astrogliosis as measured by GFAP signal on immunoblots of the soluble fraction of lumbar spinal cord (Fig. 5e).

### Pimozide Treatment Did Not Reduce Motor Neuron Loss or Levels of Misfolded SOD1 in SOD1^G93A^ Mice

**Fig. 6:**
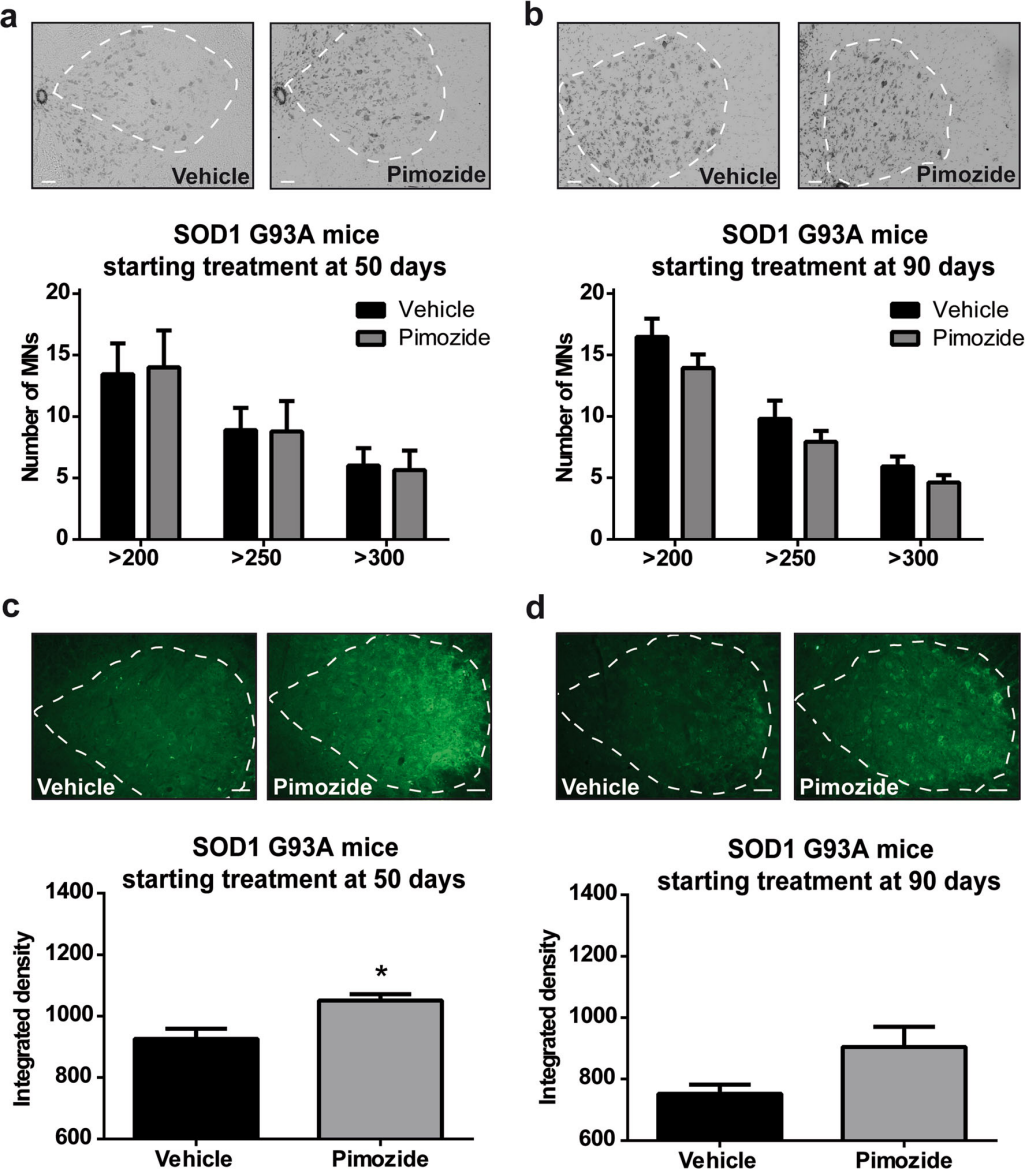
Pimozide treatment did not reduce motor neuron loss nor misfolded SOD1 in SOD1^G93A^ mice. (**a**, **b**) Representative picture of Nissl staining in lumbar spinal cord and relative quantification of motoneurons larger than 200–250–300 μm^2^ in mice at 120 days of age treated from 50 days of age (**a**) or from 90 days of age (**b**). Ten sections per mouse (*n* = 3 mice per group) were analyzed; dotted line represents area of analysis (ventral horns); data are mean ± sem and were analyzed by two-way ANOVA followed by uncorrected Fisher’s LSD post hoc test, scale bar 50 μm. No differences were observed in the number of motoneurons (two-way ANOVA analysis: interaction *p* = 0.977, motoneuron size *p* = 0.012, treatment *p* = 0.988 for treatment from 50 days and interaction *p* = 0.862, motoneuron size *p* < 0.0001, treatment *p* = 0.059 for treatment from 90 days). (**c**, **d**) Representative picture of B8H10 staining in lumbar spinal cord and relative quantification of misfolded SOD1 (integrated density of B8H10 staining) in mice at 120 days of age treated from 50 days of age (**c**) or from 90 days of age (**d**). Ten sections per mouse (*n* = 3 mice per group) were analyzed; dotted line represents area of analysis (ventral horns); data are mean ± sem, scale bar 50 μm. B8H10 signal is significantly increased in mice treated from 50 days of age (**c**) (*p* = 0.032 by unpaired *T* test) and slightly increased in mice treated from 90 days of age (**d**) (*p* = 0.104 by unpaired *T* test)

SOD1^G93A^ were analyzed for motor neuron loss and misfolded SOD1 at symptomatic stage (120 days) after having received pimozide from 50 or from 90 days of age. Microscopy analysis of spinal cord of SOD1^G93A^ at 120 days of age revealed no differences in the number of motor neurons between pimozide- and vehicle-treated mice (Fig. 6a, b). We then analyzed the levels of misfolded SOD1 at symptomatic stage (120 days) (Fig. 6c, d). Immunofluorescence staining of spinal cord with anti-misfolded SOD1 antibody (B8H10) [13] revealed an increased level of misfolded SOD1 in mice treated with pimozide as compared with vehicle-treated mice. An early treatment (50 days of age) with pimozide induced a significant increase in B8H10 signal (Fig. 6c). This was also observed with SOD1^G93A^ mice treated at 90 days of age, although the values were not significant (Fig. 6d).

## Discussion

ALS is presently an incurable disease. Pimozide, an FDA-approved compound for schizophrenia [4], delusional disorders [5], Tourette syndrome, and tics [6, 7], has recently been proposed as a new potential drug for ALS with beneficial effects at the level of NMJs [3]. Through a screening in *C. elegans* and zebrafish models of ALS and further confirmations on muscles of SOD1^G37R^ mice, pimozide appeared capable to restore motor functions in simple genetic models with positive outcomes on the structure and function of NMJs. A 6-week treatment of pimozide in a small cohort of ALS patients led to the conclusion that the drug was safe and that it improved the compound motor action potential (CMAP) of right abductor pollicis brevis (APB), but with no effects on other muscles. In November 2017, a new clinical trial (ClinicalTrials.gov; NCT03272503) has started with the aim of safety, tolerability, and clinical outcome measures on a larger number of patients. This trial involves the recruitment of 100 participants that will be treated with a single oral dose of pimozide (4 mg per day) for a total of 22 weeks [3].

Here, we have assessed the effects of chronic treatment with pimozide in two different mouse models of ALS, TDP-43^A315T^ mice, and SOD1^G93A^ mice. The duration and dosage of pimozide treatment are similar to conditions planned for ALS patients in the new clinical trial. In the case of SOD1^G93A^ mice, our pimozide treatment started at either 50 or 90 days of age until end-stage with duration of approximately 17 and 11 weeks, respectively. Since the maximum duration obtained with SOD1^G93A^ mice was 4 months (17 weeks), we decided to replicate the same treatment duration for TDP-43^A315T^ mice. In their recent paper, Patten et al. [3] used two different doses for patients, 2 or 4 mg daily, which should be equivalent to 0.35 and 0.7 mg/kg/day, respectively, in mice, according to guide for dose conversion from human to mouse [12]. In our study, we decided to administer an intermediate dosage of 0.5 mg/kg daily. After oral administration, pimozide is mainly metabolized in liver and shows a concentration peak in plasma between 4 and 12 h and a 50% loss in bioavailability for other tissues [15]. To avoid loss of drug availability, we decided to administered pimozide by intraperitoneal injection. The drug was delivered every 2 days, to reduce stress in mice due to manipulations, at the double of the concentration (1 mg/kg). There are reports showing that the drug is effective in the brain and spinal cord at 1 h after intraperitoneal injection of 1 mg/kg of pimozide with behavioral effects lasting up to 24 h [16, 17] with half-life of 55 h in the brain [8]. Moreover, subcutaneous injection of 0.63 mg/kg pimozide resulted in 30 pg of pimozide per mg of brain tissues after 8 h and 6 pg after 32 h [18]. Thus, from these previous studies, it is expected that approximately 20 pg of pimozide per mg of brain tissue would be present at 24 h after 1-mg/kg injection.

The chronic pimozide treatment performed in two mouse models of ALS resulted in no beneficial effects on behavior and pathology. In the contrary, there was exacerbation of motor performances in both TDP-43^A315T^ mice and SOD1^G93A^ mice. In TDP-43^A315T^ mice, chronic pimozide administration aggravated the loss of NMJ innervations, and there was worsening of pathological features such as increased levels of insoluble TDP-43 in lumbar spinal cord and of astrogliosis. Moreover, the chronic 17-week treatment with pimozide led to a loss of post-synaptic NMJs, an increase of misfolded SOD1, and to a shorter lifespan of SOD1^G93A^ mice.

Unlike other anti-psychotic drugs, pimozide presents advantages in the treatment of psychoses. It has a long half-life which allows it to be given to patients less frequently. It has a more specific dopamine blocking action because of its high specificity to 5-HT7 receptors which reduces the sedative effect, and it has a potent calcium-blocking activity [4]. Beneficial effects of this compound were reported in animal models. For example, *Chakrati* mice, a genetic mouse model of schizophrenia developing hyperactivity [19], showed a dose-dependent reduction of hyperactivity when treated with pimozide [20]. Neuroprotective effect of pimozide has been shown in organotypic hippocampal slice cultures exposed to oxygen glucose deprivation [21] and CA1 hippocampal neurons of rats with transient global ischemia [8]. Finally, pimozide also demonstrated anti-cancer activity by inhibiting STAT5 (signal transducer and activator of transcription 5) in cells and a mouse model of leukemia [22, 23] and STAT3 in prostate cancer cells [24].

However, deleterious pimozide-induced events must also be considered. Treatment with pimozide in schizophrenic patients can lead to cardiotoxicity and extrapyramidal side effects [4, 15, 25]. Indeed, schizophrenic patients who take pimozide have to be constantly monitored by ECG (electrocardiogram) because of the high incidence of heart rate abnormalities (QT interval prolongation) observed. Moreover, a survey on Tourette syndrome patients revealed a high tendency of increased glycemia and triglyceridemia over a period of 24 months in children taking 1–4 mg/daily of pimozide, suggesting that this molecule could be less well-suited for patients with diabetes [26]. Although a study on Huntington patients demonstrated that pimozide is able to reduce abnormal involuntary movements [27], schizophrenic pimozide-treated patients have also the propensity of receiving an additional anti-parkinsonian medication because of motor side effects, such as tremors, observed under treatment [4]. Interestingly, in the *Chakrati* mice treatment study, pimozide was the only compound inducing unusual jumping response at high doses [20]. Finally, it should be considered that pimozide is normally used in drug-induced parkinsonian tremor experiments. It can, indeed, produce parkinsonian side effects such as tremors [28–30] and tremulous jaw movements [31–33]. Hence, in our study, we also observed an increase of tremors in SOD1^G93A^ mice treated with pimozide starting from 50 days of age suggesting an early and abnormal increase of involuntary movements. Tremors of SOD1^G93A^ mice in absence of treatment correlate with early onset of symptoms [34]. Interestingly, the rotarod test performance of both animal models revealed that the negative effect of pimozide occurred rapidly after administration of pimozide. This test requires motor and coordination abilities, which involve different areas in the brain and spinal cord. The rapid drop of rotarod performance may reflect the rapid penetration and action of the drug in the central nervous system, as reported before [16, 17].

Pimozide has also been proposed as an autophagy inducer, which may have beneficial effect in neurodegeneration [35]. A study carried out with a cell system demonstrated that pimozide was able to reduce the accumulation of expanded polyglutamine repeats in a dose-dependent manner [36]. This effect is mediated by the activation of the autophagic degradative pathway, a mechanism which may reduce the accumulation of misfolded proteins [37]. In contrast, we report here that pimozide exacerbated the formation of insoluble TDP-43 in the brain cortex and spinal cord of TDP-43^A315T^ mice.

Furthermore, evidence presented in the recent study of Patten et al. [3] suggested that pimozide may confer protection of NMJs in ALS by acting as antagonist of T-type Ca^2+^ channels. On the contrary, we report here that a chronic treatment with pimozide did not alleviate proteinopathy or deficits of NMJs in TDP-43^A315T^ mice or SOD1^G93A^ mice. It should be noted that the improvements reported by Patten et al. [3] on NMJs of SOD1^G37R^ mice were based on ex vivo experiments with muscles perfused with pimozide for a short period of time, while electrophysiological parameters were recorded. In contrast, our study is based on chronic *in vivo* administration of the drug for 4-month period, and no beneficial effects of pimozide on muscle strength performance or NMJs were observed.

In conclusion, here we report that chronic administration of pimozide (1 mg/kg) in two mouse models of ALS failed to improve disease phenotypes and pathological deficits. Moreover, it is a matter of concern that pimozide exacerbated symptoms and proteinopathy in ALS mouse models when administered for a long-time period. From these results and in light of the new clinical trial for ALS with long-period pimozide treatment, we believe that pimozide should be administered cautiously to ALS patients, and a careful monitoring of patients is warranted because of potential deleterious pathophysiological effects of prolonged treatment.

## Electronic Supplementary Material

ESM 1 (PDF 1225 kb)

ESM 2 (PDF 1225 kb)

ESM 3 (PDF 1225 kb)
